# Recommendations for developing urban interventions to promote physical activity: interviews with key informants in Europe

**DOI:** 10.1080/23748834.2023.2242090

**Published:** 2023-08-15

**Authors:** Prachi Bhatnagar, Stephen Whiting, Kremlin Wickramasinghe

**Affiliations:** a Population Health Sciences Institute, Newcastle University, Baddiley-Clark Building, Newcastle upon Tyne, UK; b World Health Organization European Office for the Prevention and Control of Non-Communicable Diseases, WHO Regional Office for Europe, Copenhagen, Denmark

**Keywords:** Urban health, physical activity, interventions, interviews

## Abstract

Although there is growing evidence on what interventions can promote physical activity in urban environments, guidance on how to get these interventions implemented is lacking in a European context. To understand the process of developing urban interventions to promote physical activity, we conducted 13 key informant interviews with professionals working in urban development in five European countries, though some worked in more than one country. Participants described their experiences, challenges and recommendations across six European countries. The main challenges faced were political environments, unsupportive contexts, communication issues, working with other sectors, resource limitations and evaluations. We presented recommendations made by participants which can overcome these challenges when developing urban interventions to promote physical activity. Recommendations included obtaining cross-party political support for a project; working with local communities right from the beginning; involving all stakeholders and encouraging their commitment through identification of common goals; asking the market for solutions when faced with resource constraints, and using existing data to facilitate evaluations.

## Introduction

Physical inactivity is associated with premature mortality and prevalence of at least 25 chronic conditions (Lee *et al*. 2012). Up to 8% of deaths globally are attributable to physical inactivity (Katzmarzyk *et al*. 2022). There are many determinants of physical activity (Sallis *et al*. 2006), but previous research has demonstrated that features of urban environments, such as the number of parks and density of public transport networks, are consistently and linearly associated with physical activity levels (Sallis *et al*. 2016). Cities are well placed to improve physical activity because they are large enough to act on the determinants of physical activity behaviour, but small enough to understand the needs of their local populations (Borrell *et al*. 2013).

In Europe, over 74% of the population currently lives in urban areas, with this expected to increase to over 80% by 2050 (United Nations Department of Economic and Social Affairs 2018). With urban populations increasing, we need to create equitable, healthier cities to meet the targets of the United Nations Sustainable Development Goals (SDGs). The European Healthy Cities network has facilitated progress towards this goal (World Health Organization 2019), contributing to a wealth of information on the areas that cities need to act on to improve health (World Health Organization. Regional Office for Europe 2022). However, there remains much more to do if the SDGs are to be achieved over the coming decade.

To make more rapid progress towards achieving the SDGs, we need to be working on the most useful activities necessary for modifying urban environments to promote physical activity. This includes knowing not just *what* urban interventions to focus on but also *how* to go about getting these changes implemented successfully. Previous studies have explored the process of designing physically active communities (Rydin *et al*. 2012, Bergeron and Lévesque 2014, Le Gouais *et al*. 2020, Firth *et al*. 2021). However frameworks have been developed from a Northern American context (Bergeron and Lévesque 2014, Firth *et al*. 2021); while some findings will translate to a European context, the urban context of Europe is undoubtedly different to that of Northern America. Others have provided very broad recommendations (Rydin *et al*. 2012) or in the other direction, have had a very specific focus on the use of evidence in local planning (Le Gouais *et al*. 2020).

To provide guidance for stakeholders interested in making changes to urban environments to improve health, we aimed to understand more about the process of creating urban interventions to promote physical activity in the WHO European Region. Specifically, we were interested in learning from professionals who have already developed urban interventions that promote physical activity to answer the following research questions 1) What steps are commonly undertaken? 2) Which stakeholders do they involve? 3) What barriers have they faced? and 4) What has been helpful for them when developing urban interventions?

## Methods

### Study design

As we were predominantly interested in process, we used qualitative methods to address our research questions. We conducted semi-structured interviews with 13 key informants. We used Framework Analysis to analyse the interviews, which is particularly suited for policy research and for research with short timeframes (Gale *et al*. 2013).

### Participants

The 13 participants were from a range of backgrounds and came from five European countries. Job descriptions or titles provided by the participants included academic, architect, city councillor, department of city development, director of a sports-related charity, healthy cities expert advisor, international affairs strategist, public health consultant, traffic planner and urban designer. Participants were based in Denmark, Hungary, Portugal, The Netherlands and the United Kingdom. Recruitment was conducted through a) three emails to the WHO Healthy Cities Network inviting people to share their experiences of developing urban interventions to promote physical activity, b) eleven emails to potential participants identified through published books and articles, and c) through snowballing, where we asked participants to let us know of any other people they thought we should contact. Of the eleven potential participants who were sent an email inviting them to participate in the study, four did not respond. Our criteria for recruiting people were that participants had to have first-hand experience of developing, implementing or evaluating urban interventions that promote physical activity. The original intention of this project was to provide guidance to the WHO within a short time-frame, therefore we recruited as varied a sample as possible within the timeframe available to us.

All interviews were conducted in English by the lead researcher. Ethics approval was received from the University of Oxford Central University Research Ethics Committee (CUREC) Approval Reference: R66719/RE002.

### Data collection

The topic guide was developed in conjunction with the co-authors from World Health Organization. We started with an easy introductory question where the participant could talk about their job role, covered the specific research questions the WHO wanted to answer around process of developing and evaluating these types of projects, and also included a general open-ended question to allow for topics we had not anticipated as being relevant. Specifically, the topic guide covered questions on describing an urban intervention they had been involved with that had a primary or secondary aim to increase physical activity, how the described project or policy was initiated, who was involved in the project, what challenges were faced, what was helpful and what recommendation participants had for others wanting to develop urban interventions to promote physical activity (see supplementary material for the topic guide). The main elements of the topic guide were used as the initial broad themes for analysis.

The lead researcher conducted 12 interviews through remote conferencing software such as Zoom, Skype or Microsoft Teams. Interviews lasted between 30 and 90 minutes, were audio-recorded and professionally transcribed. One participant preferred a written interview and emailed their answers in response to questions we emailed them.

### Data analysis

Using a Framework analysis approach (Gale *et al*. 2013) allowed us to be focused in our analysis of the data, allowing both for finding data for our pre-existing themes and for new themes to emerge from the interview data collected.

Transcripts were checked for completeness by the lead researcher. All analyses were conducted in NVivo 12. We began with two researchers doing open coding of the first two transcripts in order to develop an analytical framework to apply to the remaining transcripts; the broad themes from the topic guide were used as overall categories, with the open coding aiming to provide some sub-themes within these. The two researchers compared their codes and came to an agreement on an analytical coding framework through resolving disagreements through dedicated discussion over how we had come to different codes and exploring where we had overlap. Once developed, the lead researcher read the remaining transcripts to apply the codes from this analytical framework. New codes were added to the analytical framework if appropriate; the previous transcripts were re-read to see if this new code was relevant. We used NVivo to generate a framework matrix of participants against the main themes. We summarised the data for each theme and participant. The lead researcher wrote memos as they coded the data to document and process thoughts on the codes and potential further analysis. After analysing the 13 interviews, we considered whether we were likely to see more themes in the data with further participants, weighing this against the short time-frame we had available. Given that all participants had described similar challenges and we had seen recommendations to overcome many of these, we decided to stay with the 13 interviews.

## Results

We asked all participants to describe an urban intervention they had been involved with that had a primary or secondary aim to increase physical activity. Some participants described policies that had been developed, while others described specific urban modification projects; projects and policies spanned six countries (Table 1).

**Table 1.** Summary of projects described.

Country	Description
**Policies**	
Portugal	Bringing culture to all parts of the city through facilitating dance, sport, youth and music events in parts of the city where these events are not traditionally held.
Portugal	Making urban parks more of a focal point for local communities through encouraging local groups to use parks to hold local events such as music and yoga.
Russia	Shifting a city’s transport infrastructure away from car-based to tram, cycle and pedestrian-based.
The Netherlands	Making health central to all policy decisions for the city to stimulate healthy behaviours
The Netherlands	Increasing the quantity and quality of green space in a neighbourhood
United Kingdom	Increasing the amount of bike storage that housing developers have to legally include in the homes they build.
United Kingdom	Embedding a healthy streets framework into a city’s transport sector.
**Urban modification projects**	
Denmark	Addition of cycle lanes or pavement networks to improve the active transport network
Germany	Developing urban parks
The Netherlands	Redesigning a bus station to also consider the pedestrian network connecting people to the bus station
The Netherlands	Removing a highway to restore an old canal
United Kingdom	Improving the quality of an existing pathway which is near a lot of employers and has a lot of potential to link areas together.

Participants then discussed the process they had gone through to develop these policies/projects, talked about the challenges faced and recommendations they had for other people wanting to develop similar projects/policies. This paper focuses on the recommendations made by participants, categorized according to the six main categories of challenges discussed by participants (Table 2). Supporting quotes for the challenges described by participants are in the supplementary material.

**Table 2.** Challenges and recommendations when developing an urban intervention to promote physical activity (see supplementary tables for supporting quotes).

Challenges Described	Participant Recommendations (blank indicates no recommendations noted)
**Politics**	
Political agenda superseding the evidence base	Get politicians on side by using rhetoric that does not alienate any particular political group.
Getting long-term political buy-in	Go straight to the top of the political chain
**Unsupportive contexts**	
Valuing economics over health	Work with, not against economic development - advocate to bring health into it.
A historical focus of planning around cars	Long-term lobbying from a range of partners
**Communication**	
Getting public support for projects	Talk with, not at communities
Understanding local need	Work with communities from the beginning of the process Learn about local needs
Public discontent about infrastructure disruption	Have a good PR campaign to gain public support
**Working with other sectors**	
Learning the language of a new sector	Use language understandable by all sectors
Getting commitment from stakeholders	Find common goals such as equity to bring sectors together Develop personal relationships Develop a governance system All stakeholders involved from the beginning
**Resource limitations**	
Limited governing powers at a local level (context dependent).	When faced with multiple tasks, pick the most impactful ones Ask the market for solutions Use each other’s strengths
Budget constraints	Focus on broader visions to sell to politicians Consider starting with a smaller, or easy to sell project.
Missing data on pedestrian and transport networks	
Losing knowledge about previous projects done in the area	
**Evaluations**	
Difficult to get external funding	Use existing tools and dataTrust existing evaluation tools Trust existing evaluation tools
Conducting evaluations or impact assessments too late	Do evaluations and impact assessments at the beginning or throughout
Using appropriate evaluation methods	

### Politics

Some of the interventions described were actually political strategies, and others were specific urban modifications. In both cases, participants viewed the changeable nature of politics as a risk to the success of the project. The challenge of the political agenda superseding the evidence-base for an intervention was also described. To overcome these issues, participants recommended going straight to the top of the political chain to get cross-party political support to ensure the project was not dependent on the current political administration.

I think, yes, I would go straight to the top of the politics. So, again, the party leaders and the mayors, and the chairs of committees, or whatever they have. Get to the parties that - and not just one party, but try and draw them together. - Emeritus Professor of planning, health and sustainability

Using language and rhetoric that does not alienate any political side was given as a specific recommendation to achieve this; an example given was on how the value of ‘freedom’ can be perceived differently by left and right wing political groups, presenting an opportunity to use a concept that could bring people together.

So you have to – to my mind, you’ve got to orientate the rhetoric so that it doesn’t alienate any particular political – powerful political group, which might come into power. And so, for example, the rhetoric of freedom is associated with right wing oppositions. But if you write freedom in a different way, freedom for all, rather than simply freedom, then it becomes more of a socialist thing, freedom for all … - Emeritus Professor of planning, health and sustainability

### Unsupportive contexts

Participants described a trend of valuing economics over health within the political field. To overcome these challenging contexts, it was recommended to aim to shape broader economic policies rather than fight against them. For example, to work with economic development by bringing health into the discussion.

… you leave it to market forces to just create their own logic, or you try and shape those market forces. Not go against them because that’s not sensible, but you have to shape them and create the market that is going to be good for health, good for carbon reduction, and so on. - Emeritus Professor of planning, health and sustainability

Related to this, a historical focus on planning around cars was also problematic for getting support within the political arena. Long-term lobbying at national and international levels against car-use in urban areas was recommended as a long-term strategy to overcome this challenge.

But the system that the transport department are working in is one that is set at a national and an international level, which is that we have an oil based economic system, we have a manufacturing base in building cars and because of this, for the last 50 years, everything has been set up to make it easier and easier for people to drive cars. And if we want to unpick that system, which is what we actually have to do if we’re going to increase physical activity, in terms of people walking and cycling, it needs the kind of long term lobbying from a range of partners against cars and car use in urban areas, rather than advocacy at a much lower level for promoting walking and cycling. - Public Health Consultant

### Communication

Obtaining community support from the beginning and understanding the desires of the local community overcomes the dual challenges of making sure that local needs are met, and that the public does not misconstrue any implemented policies or projects.

Several participants described working with local communities as a key step for starting successfully. There were different methods of engaging with communities, however all those who described this recommended early and regular communication with the communities in question. One participant’s recommendation to *‘talk with people, not at them’* encapsulated this idea well. This early communication also ensured that any urban project meets local needs.

But obviously, what’s actually better is by having the consultation and making it as transparent as possible, right at the beginning of the process … .and so, well that’s why we often say, it’s just so much better to know about the big obstacles at the beginning, rather than when you’ve spent a whole load of money and then have to try and think how to overcome the problems. - Architect

As at the beginning of a project, continued community support is important for project success. As modifying urban environments necessarily creates disruption over months or even years, communicating with the public while disruptions to infrastructure are happening is vital. For example, one participant described a project where they had provided a new route planner to help people navigate the fastest route while infrastructure changes were underway.

So we created our own route planner, just as Google has, but our route planner was including all the day to day closuring, one way streets, and all that, it was all in the model. So you could say where you go from A to B and then we could propose your route for today, tomorrow, or whatever day – what day you need it for, and then we could give you alternatives and say, well if you don’t take the car you can do it faster on a bike using this route and doing it this way. So it was a more personal, a more precise route planner, it was very popular and very well used. – Traffic planner

### Working with other sectors

Understanding who the full range of stakeholders are was seen as vital to starting a project successfully. Stakeholders vary according to the project or strategy being developed, therefore participants described a large variety of stakeholders (Table 3). Having this information at the beginning of the project enabled stakeholders to be involved early and throughout, facilitating a successful implementation.

And the architects and urban designers were producing the scheme, which will then come to a meeting, which we organised, which had in it local councillors, it had the owners of the site, it had the developers, the potential developers of the site, it had the designers, it had the local planning authority, it had local people, as it were, delegated from a big meeting we held first of all, delegated down through a focus group, so that everyone was there together, and then a particular process of engagement and mutual discussion, to ensure that people started to understand their different points of view. - Emeritus Professor of Planning, Health and Sustainability

**Table 3.** Stakeholder groups mentioned by participants.

Category	Stakeholders
**Politics**	Politicians
	Local government
	Public health
	Policy officer
**Urban Planning**	Architects
	Developers
	Green space managers
	Property agents
	Traffic engineers
	Planning sector
**Academia**	Local university
**Public**	Parents
	Residents
	Local business owners
**Finance/Legal**	Accountants
	Legal sector
**Communications**	Dedicated communications team
	Local press
**Third sector**	Environment
	Other relevant civic groups

Some participants provided useful insights into how they had successfully collaborated with people from sectors different to their own. Finding common goals and language, developing personal relationships and having a governance system were all discussed as important for a successful working relationship with other sectors.

Whereas what is actually effective is learning their language and their priorities and helping them through the way that they do things to get to the outcomes that we want to see. So rather than trying to win them over it’s about, how do you gently steer them through what they’re doing towards a shared benefit? - Public Health Consultant

Finding a common goal has the potential to overcome the challenge of obtaining time commitments from people in different sectors. The use of equitable urban spaces as an argument to bring people together was seen as a valuable strategy. One participant described how this realisation of equity as a desirable shared outcome had come from a department they were collaborating with.

And after a few years, my colleagues from the welfare department said there’s also a very social aspect in this strategy as well, and we missed that. And we want to be more involved in that part as well. And at that time, we had all these discussions about equity in cities, and so, we said yeah, actually, it’s not healthy urban living, but it’s healthy urban living for everyone. - International affairs strategist

The broader focus on equitable streets was also a helpful strategy when working with colleagues who are only passionate about very specific types of physical activity, such as cycling or sports.

I find sometimes that the people who choose to work on physical activity or on walking or cycling are people who are personally passionate about cycling or fitness and it’s – can be quite unhelpful, because what you want are people who are genuinely passionate about making streets more equitable for people rather than people who just love cycling. – Public Health Consultant

However, going beyond the identification of shared objectives was also recommended. One participant who had successfully worked with another sector described the importance of developing personal relationships with colleagues from other sectors, and not just relying on the evidence-base to change their priorities.

And a lot of it is about not making reasoned evidence based arguments, which is the fallback for the public health community. But really what means people do their jobs differently is politics, personal relationships, a sense of agency over what they’re doing, a sense of pride in what they’re doing, having the right tools and training available … but I definitely learnt on day one that talking about evidence base gets you pretty much nowhere. - Public Health Consultant

Participants also described the challenges of learning the language of a new sector. The recommendation to overcome this was to avoid using jargon and focus on language that is comprehensible by all sectors.

So the success of this framing is that it’s not in a language of public health, or in a language of transport, it’s in a plain language that is quite apparent to a wide range of the different sectors. They can look at it and go, if they do that, that will achieve my objective so I’m happy to endorse that. – Public Health Consultant

Finally, one suggestion to overcome the challenge of getting time commitments from colleagues in different sectors was to have a governance system to discuss linked projects within the same meetings, so as not to exhaust staff.

So, rather than, you know, people having to keep going to different meetings, there was just one meeting … So, straight away you had a ready-made, sort of, governance system, and I think that’s very important, to make sure you’ve got that governance system. If there isn’t one in existence, you have to set it up … And because there isn’t a vast army of all these specialists, you know it’s going to be the same people over and over again. - Traffic planner

### Resource limitations

Multiple participants described issues with financial or budget constraints. Challenges included funding for stakeholders to participate in a project and not having the budget for the desired project. Two participants’ recommendation to overcome this was to sell ideas to politicians by focusing on a broader vision for a city or urban network, rather than on specific projects.

… most often in public administration you look for the money you have and then you say, well we cannot afford a bridge, or we can only afford a very poor one or unpleasant, and so on, and then you use the money you got. But you can turn it the other way round and have it as part of an urban development, and then it’s to create visions and ambitions for our politicians, and then they sometimes manage to get the budget for that. – Traffic planner

However, starting a large project with a smaller, or easier to sell venture was also helpful in getting initial support. This was also said of larger urban interventions that had the potential to pave the way for further investment into a policy or large project.

we always think in terms of short, medium, and long term things. And the short ones, which we sometimes call, quick wins, is to have cheap and quick interventions being made that start to show people that things are starting to change, and whether it’s some benches somewhere that don’t get vandalised, you know- Architect

And so they inserted the tram system into the heart of the city and they pedestrianized and created cycle routes in the heart of the city. And because pedestrianisation of that was politically easier to do they showed, as it were, showed what was possible. And then basically built out from that with progressive investments in low energy transport, which means walking, cycling, and public transport- Architect

Local governments working at the city level are working within a legal and political context that is set nationally. Depending on the context, this may result in limited governing powers at a local level. In these circumstances, innovative approaches may be needed, with one recommendation to ask the market for solutions and use each other’s’ strengths.

… don’t do everything on your own, but try to use each other’s strengths, as well. And don’t be afraid to ask the market for solutions as well. That was something, one of the best, those bus stops, we were all so excited about it, and it, but particularly because it wasn’t our own idea. - International affairs strategist

Time was also a resource that is in short supply, often due to multiple demands on time. Picking the most impactful tasks to achieve your goal was recommended as a strategy to deal with this.

… people come to you with this long shopping list of things that they want you to do, and you have to – I mean, this is true, I guess, in any job, but you have to stick quite doggedly with doing the few things that you think are going to make a really significant difference, and not get distracted by the things that other people ask you to do. And sometimes when people don’t want you to rock the boat too much they will go and ask you to do something that they know will keep you out of the way for a while … So it’s picking … asking themselves critical questions about, is this really the best use of my time and am I really being impactful here? – Public Health Consultant

### Evaluations

Urban modification projects often require environmental or health impact assessments – what is required will vary according to the country and its own regulations. It was seen as important to get these done at the start of the project, rather than halfway in, or towards the end. This minimises unnecessary modifications, and means that the information gained from the assessment can actually be incorporated into the project design. Evaluating throughout a project was also recommended to make the most of evaluation findings.

To be very succinct about it, end point evaluations have limited value. They – you might mitigate all the problems but you won’t tackle the essence of it. While if you can integrate the process of evaluation right the way through then you’ve got a real chance of making a good plan, or a good project. - Emeritus Professor of planning, health and sustainability

It was acknowledged that, for academics, it can be difficult to obtain funding for rigorous evaluations that are incorporated throughout a project. To facilitate the process of evaluation, participants recommended using validated evidence-based tools and data that are already being collected. Participants referred to existing surveys or routinely-collected data, but also pointed to the possibility of developing new approaches to take advantage of smartphone data.

… try to use available data that’s being collected, either as – I mean, there’s more and more data collected that’s available through things such as, GP practice data, so doctors prescriptions and things like that, that can help looking at before and after, so taking advantage of existing data sources. Or developing approaches so that we can obtain data from individuals on their behaviours through smartphone apps. - Academic

### Remaining challenges

Participants discussed missing data and the use of appropriate evaluation methods as specific challenges for developing and evaluating urban interventions that promote physical activity. However, they struggled to give recommendations to overcome these.

Missing data was a challenge for some participants when developing or evaluating projects. There were difficulties working with the local authority where there had been staff changes, but no retention of knowledge about projects that had already been done; this resulted in one participant being asked to develop a similar project twice within a few years. Missing data on pedestrian networks was also cited as a difficulty, not only for evaluating a project, but also for conceptualising the impact of an intervention on pedestrians.

But there is so many – there is so much knowledge still missing that you cannot really make cost benefit analysis for walking. It’s pretty difficult because a lot of things are just not – we don’t have any figures about it, or we know there is an effect but we don’t know how big the effect is. Let alone that we can say what the effect is in Euros or – and so I think a lot of the benefits of walking are just a little bit too vague, so you cannot really count them. For example, like the social benefits of walking, including people, or people will be less lonely if they meet people on the street. How are you going to measure this? That’s quite difficult. -

One participant also described the challenges of evaluating health outcomes from systems-level interventions. They described the medical model with a distinct causal pathway as being inappropriate for urban interventions are that are acting in a causal network; the complex nature of interventions means that contributing how an urban intervention has affected physical activity is not always possible.

… because you’re moving from a closed system and a very distinct causal pathway, in terms of medicine, to what I would call a causal web or a causal network, and because – yeah, you’re moving from a reductionist science to a holistic science, system science … I mean, you can have an outcome measure, how many houses in Bristol can store more than one bike per dwelling? You know, you can have very simple project outcome measures, but to track that right through into health of the population, or increased physical activity of the population, is almost something you – shows that you – thinking about it in the wrong way. - Healthy cities expert advisor

## Discussion

### Key findings

We have reported recommendations for encouraging successful urban intervention projects or policies to promote physical activity. Key recommendations for starting successfully included ensuring political support, working with the local community, ensuring all relevant stakeholders are involved and doing any impact assessments early. During the project, participants described strategies for effective multisectoral working, dealing with resource constraints and continued communication with the public. Evaluations were recommended to be done throughout, use existing data and to be designed using appropriate frameworks such as a systems approach.

### Findings in the context of existing research

The requirement to have political support to successfully carry out city-level projects that increase physical activity is in line with findings from previous research. The 2017 WHO report on increasing physical activity in cities acknowledges the need for urban projects to have political support commensurate with the long timescales of such projects (World Health Organization 2017) and was one of the five recommendations from The Lancets’ commission on Shaping Cities for Health (Rydin *et al*. 2012). Our study confirms the finding by Bergeron and Lévesque (2014) where political buy-in was ranked as a feasible and important concept, further strengthening the argument for gaining cross-party political support (Bergeron and Lévesque 2014).

We begin to build on this with a specific recommendation of using choosing appropriate political language in order to achieve this. For example, when discussing concepts that can be palatable across the political spectrum, frame these in a manner which will be appealing to the audience you are speaking to; in our study, a participant used the concept of freedom as a specific example.

Our findings confirm the importance of a community engagement process to determine local needs, reported in previous research (Bergeron and Lévesque 2014); although not discussed by our participants, having the support of the community for an intervention is also likely to facilitate getting political support. However, we go further to suggest that not just engagement but co-design of interventions may also be valuable. Engaging the public in the design or planning process was not ranked highly in Bergeron and Levesque’s study (2014), however qualitative work in a recent evaluation of an urban green space intervention found that the lack of community involvement in the design of the intervention meant the intervention was perhaps not appropriate for increasing physical activity in the target group (Benton *et al*. 2021). We suggest that further exploration into feasible and appropriate methods of co-creating urban interventions with local communities may be necessary.

As found in our study, stakeholder collaboration and buy-in emerged as a key theme for successful built interventions in a concept mapping exercise conducted in four Canadian cities (Firth *et al*. 2021) and in the study by Bergeron and Levesque (Bergeron and Lévesque 2014). The importance of engaging stakeholders early and throughout is also highlighted in 2013 study that explored the key elements for success for community-based physical activity interventions (Haggis *et al*. 2013). We provide a themed list of possible stakeholders mentioned across all projects, which could provide a good starting point to think about all aspects of the project; for example, consulting local universities may help with evaluation activities. Nonetheless going through a process of stakeholder mapping is key to ensure that each project has the appropriate sectors or groups involved from the beginning.

Previous research has explored successful ways to achieve working with different sectors (Kuruvilla *et al*. 2018, van Dale *et al*. 2020, Mondal *et al*. 2021). Many of the recommendation made by these papers overlap with our findings on multisectoral working. Kuruvilla *et al*. (2018) who synthesized findings from case studies in maternal and child health recommended framing problems at higher level that relevant to all the sectors involved and the public good; our participants’ suggestions of using equity arguments to find a common goal provides a concrete example of this in the physical activity and built environment field. These studies also point to the challenges of funding multisectoral collaborations, having sufficient resources and the importance of regular evaluation throughout a project (Kuruvilla *et al*. 2018, van Dale *et al*. 2020). Our participants’ recommendations to overcome budgeting and resource challenges included selling broader visions rather than projects, and starting with smaller practical elements of a project to gain confidence.

Previous papers have been published that make recommendations on the use of evidence in built environment interventions (Le Gouais *et al*. 2020). Enabling the appropriate use of evidence when developing interventions is essential, however our findings imply that other strategies also need due consideration when convincing and working with multiple sectors. Personal relationships and steering discussions in a favourable direction emerged as important for multisectoral working in our study; this finding seems to be confirmed in a meta-narrative review conducted by Mondal *et al*. (2021) where they discuss the importance of building trust and being able to navigate complex communication.

Our participants reported that was that is difficult to obtain appropriate funding for evaluation, or that it is common to attempt to capture the distal effects of a built environment intervention on total physical activity levels. Our findings that there is a mismatch between evaluation approaches and the appropriate theory was interesting given the published literature on the importance of a systems approach for physical activity and public health interventions (Kohl *et al*. 2012, Rutter *et al*. 2017). Although the published research evidences that these discussions are happening within academia, our findings suggest that current thinking on evaluative approaches may not have adequately filtered through to funding bodies and/or people working at the local level.

Although not discussed by our participants, measuring the appropriate outcomes is also a challenge for evaluation design as appropriate data need to be available and able to be analysed at the appropriate time points. This ties in with one participant’s point about evaluation needing to be thought about from the beginning of the project, but also other participants’ recommendation for the need for co-design of interventions with stakeholders. Although universities were on the list of stakeholders mentioned by participants, other stakeholders relevant for collecting evaluation data could also be considered as integral to designing urban interventions that promote physical activity, perhaps considering more innovative data sources (Bannan *et al*. 2022).

### Strengths and limitations

Our focus on the process means that we were able to provide some insight into the practical steps taken to develop urban interventions to promote physical activity. We also aimed to go beyond describing the barriers to developing built environment, or urban interventions to promote physical activity, but to present a set of recommendations to overcome these challenges, given by respondents.

Due to the time constraints of the project, we were unable to recruit more professionals with varied job types, or further participants with the job types already included. Doing this may have yielded further themes that did not emerge in our study, but we are confident that the major themes are present. All interviews were conducted in English which is likely to have affected recruitment. We were recruiting during the COVID-19 pandemic and relied on emails to the WHO Healthy Cities network and published authors to recruit participants. However, we may have recruited more people though attending in-person conferences/meetings and through consideration of other networks associated with cities, or the different professionals we interviewed, such as architects and town planners.

The countries that professionals worked in and the countries where projects were carried out are predominantly based in Western and Northern Europe. The contexts of the countries in these areas of Europe vary between each other, and also vary from other parts of Europe such as Eastern Europe and the Mediterranean.

One aim of this paper was to find general principles professionals have followed in the process of designing urban interventions that promote physical activity. However, it should be acknowledged that our general recommendations need to be considered alongside the local political, social, financial, cultural and historical context (Craig *et al*. 2018) of any city wanting to implement these recommendations. By taking these aspects into consideration during design and evaluation we have the possibility of understanding how the intervention works or does not work, and who it works for. Taking into account local context also allows for exploration into what aspects could be successfully adapted, scaled up or translated from one context to another and how far effects observed in one context can be generalised to another.

## Conclusion

We have provided specific recommendations to overcome the common challenges faced by people wanting to develop urban interventions to promote physical activity. Due to the long-term nature of urban interventions, getting engagement from local communities and politicians across the spectrum right from the start is key. Recommendations for evaluation activities, the realities of working with other sectors and dealing with resource constraints are also detailed.

The frustration expressed with inappropriate evaluation techniques and lack of appropriate funding indicates that the use of natural experiments and mixed-methods techniques may need wider dissemination to funding bodies and at the regional level of governance. We also recommend further research into whether the processes describe here are appropriate to other parts of Europe, further exploration into the successful methods of working with communities to develop built environment interventions, and an assessment of the evidence-base on whether interventions co-designed with the local community are more effective at increasing physical activity levels than those that are not co-designed.

## Supplementary Material

Supplemental Material
