# Supplementary material for: Recommendations for developing urban interventions to promote physical activity: interviews with key informants in Europe
Source: Cities Health. 2023 Aug 15;7(6):1002–11. doi: 10.1080/23748834.2023.2242090 (PMC10591599; doi:10.1080/23748834.2023.2242090)
Supplement: Supplemental Material [file RCAH_A_2242090_SM4029.pdf]

Supplementary table: Topic guide

**Question**	**Prompts**
**PARTICIPANT JOB ROLE**	
Can you tell me about yourself?	
**PROJECT DESCRIPTION**	
We are here to discuss environmental interventions to promote physical activity. Can you tell me about a project you were involved in?	*What was the main aim of the project?*<br><br>*What was the intervention/project?*
**PROJECT BEGINNINGS**	
How did the idea for the intervention come about?	
**STAKEHOLDERS**	
Who was involved in developing the intervention?	*If don't mention, ask them what their role was*<br><br>*Were there any other sectors or organisations involved?*
How did you approach these people or organisations?	
At what point did you approach them?	
**CHALLENGES**	
What challenges did you face developing this intervention?	
**FACILITATORS**	
What was helpful when developing the intervention?	*What went well?*
**EVALUTATIONS**	
Do you carry out any evaluation activities for these projects?	
**RECOMMENDATIONS**	
What advice would you give to other people wanting to develop environmental interventions to promote physical activity?	
**ENDING**	
Is there anything else that you would like to tell me?<br>Thank participant for their time	

Supplementary table: Challenges described by participants

**CHALLENGES DESCRIBED**	**SUPPORTING QUOTES**
**Politics**	
Political agenda superseding the evidence base	*"…and then the Mayor made a political decision on the matter, which was unrelated to the evidence presented…"*<br>*Public Health Consultant*
Getting long-term political buy-in	*"… politics can be very disruptive because you have one bunch in control and then there's an election and the others get into it. And typically the first thing the new ones do is get rid of whatever had been proposed by the old ones, because they don't – they want to show that they weren't – that it was unpopular in whatever way. "*<br>*Architect*
Unsupportive contexts	
Valuing economics over health	*"Well primarily, I suppose, in Britain at present, because the government rules the planning policy and … which basically favour the market and so it's difficult for local authorities to combat that…. Who's going to make money out of it? All these things come into it inevitably."*<br>Emeritus Professor of planning, health and sustainability
A historical focus of planning around cars	*"…it may be what the transport policies have been for the last three decades or… since the War really, which have all tended to be centered around easing traffic flow rather than promoting healthy urban – healthy physical activity through active travel and recreation, and so on. So the context within which the decisions are being made, the strategic context, is difficult to combat and requires almost going back to square one."*<br>Emeritus Professor of planning, health and sustainability
Communication	
Getting public support for projects	*"But one thing that you tend to do when you are in a power position, is patronising, you know, is talking to people saying, "Look, this is what you should do." That's the wrong thing to do, you know? …. So we're here to cooperate, not teach, you know? Not have a pedagogical attitude. So yes, that's one of the challenges."*<br>City Councillor
Understanding local need	*"I'm always telling to everyone I advise about physical active public space, physical active friendly environments, please please please please think about if you going to do this how it's going to be used."*<br>Academic
Public discontent about infrastructure disruption	*"When you do this kind of project, first you get all the negative sides, you get all the road closures, detours, noise, construction work, and all that. And then, many years later, you get all the benefits."*<br>Traffic Planner
Working with other sectors	
Learning the language of a new sector	*"Yeah, I mean, I think people complain a lot about not understanding each other's languages, and I sometimes feel that in some conversations that we have… But because people have different objectives, they have different things that they want to get out of the process, it's not always*

	easy to understand the other person's perspectives of what they're trying to achieve."<br>Academic
Getting commitment from stakeholders	*"Well, of course, time is always – finding people that are committed enough to find the time to make – to join all these different types of events is difficult. …so it tends to go down the drain….."*<br>Academc
Resource limitations	
Limited governing powers at a local level (context dependent).	*" Well unless the central government gives, or allows, powers to local governments to do that then of course it's very difficult. Government, not only shapes the messages it gives but the actual legal context within which local authorities operate is going to be very very important. "*<br>Emeritus Professor of planning, health and sustainability
Budget constraints	*"And yeah, what actually happened, the reason there was a long delay was that a variety of other ideas came flying along to do with financial reasons, ..and they, essentially, then that's why there was a lot of time that got spent of various other options being suggested, sometimes being actually quite different from what we proposed at the event"*<br>Architect
Missing data on pedestrian and transport networks	*" So – and that's, I think, there are very few municipalities who have a vision about a total route network of pedestrians. So I think that's a problem – actually, I think there are a lot of municipalities, if you would ask them, can you give me a map of your pedestrian route network, that they will not be able to, because they just don't have it. "*<br>Urban designer
Losing knowledge about previous projects done in the area	*" I have to tell them that I already did a project there, actually two, and if they say, okay, right, well this – well the people you then worked with they do other things now, so now we have a new team and well maybe you can send us the things you did two or three years ago. And I'm, like, okay, so you don't have any way to – I mean, can't you just look at a digital, I mean, at a map somewhere, like, okay, here are all the reports done on this area in the past, I don't know, five/ten years, yeah, well bit difficult, bit difficult, easier if you just send them to us."*<br>Urban designer
Evaluations	
Difficult to get external funding	*"it's really finding a way to be flexible with the funding so that you can adjust/adapt, just – but that's – the key thing is, I don't know how you – I don't know how to make that happen with the funders to make sure that there's that flexibility that's integrated within the process, plus figuring out ways to be flexible and adaptable to whatever the circumstances, and changing circumstances."*<br>Academic
Conducting evaluations or impact assessments too late	*"Now the conclusions of that was that these various appraisal techniques didn't have very much impact on the actual content of plans and policies and project - didn't have very much impact. That's because they were often done too late in the process rather than being, simply, the process, part of the process."*<br>Emeritus Professor of planning, health and sustainability
Using appropriate evaluation methods	*" I mean, you can have an outcome measure, how many houses in Bristol can store more than one bike per dwelling? You know, you can have very simple project outcome measures, but to track that right through into health of the population, or increased physical activity of the population,*

	is almost something you – shows that you – thinking about it in the wrong way."<br>Healthy cities expert advisor

Supplementary table: Recommendations described by participants

PARTICIPANT RECOMMENDATIONS	SUPPORTING QUOTES
**Politics**	
Get politicians on side by using rhetoric that does not alienate any particular political group.	"So you have to – to my mind, you've got to orientate the rhetoric so that it doesn't alienate any particular political – powerful political group, which might come into power. And so, for example, the rhetoric of freedom is associated with right wing oppositions. But if you write freedom in a different way, freedom for all, rather than simply freedom, then it becomes more of a socialist thing, freedom for all…" - Emeritus Professor of planning, health and sustainability
Go straight to the top of the political chain	*"I think, yes, I would go straight to the top of the politics. So, again, the party leaders and the mayors, and the chairs of committees, or whatever they have. Get to the parties that - and not just one party, but try and draw them together." - Emeritus Professor of planning, health and sustainability*
**Unsupportive contexts**	
Work with, not against economic development - advocate to bring health into it.	"…you leave it to market forces to just create their own logic, or you try and shape those market forces. Not go against them because that's not sensible, but you have to shape them and create the market that is going to be good for health, good for carbon reduction, and so on." - Emeritus Professor of planning, health and sustainability
Long-term lobbying from a range of partners	"But the system that the transport department are working in is one that is set at a national and an international level, which is that we have an oil based economic system, we have a manufacturing base in building cars and because of this, for the last 50 years,

	everything has been set up to make it easier and easier for people to drive cars. And if we want to unpick that system, which is what we actually have to do if we're going to increase physical activity, in terms of people walking and cycling, it needs the kind of long term lobbying from a range of partners against cars and car use in urban areas, rather than advocacy at a much lower level for promoting walking and cycling." - Public Health Consultant
**Communication**	
Talk with, not at communities	*"talk with people, not at them" – City Councillor*
Work with communities from the beginning of the process<br>Learn about local needs	*"But obviously, what's actually better is by having the consultation and making it as transparent as possible, right at the beginning of the process….and so, well that's why we often say, it's just so much better to know about the big obstacles at the beginning, rather than when you've spent a whole load of money and then have to try and think how to overcome the problems." - Architect*
Have a good PR campaign to gain public support	*"So we created our own route planner, just as Google has, but our route planner was including all the day to day closuring, one way streets, and all that, it was all in the model. So you could say where you go from A to B and then we could propose your route for today, tomorrow, or whatever day – what day you need it for, and then we could give you alternatives and say, well if you don't take the car you can do it faster on a bike using this route and doing it this way. So it was a more personal, a more precise route planner, it was very popular and very well used." – Traffic planner*
**Working with other sectors**	
Use language understandable by all sectors	*"So the success of this framing is that it's not in a language of public health, or in a language of transport, it's in a plain language that is quite apparent to a wide range of the different sectors. They can look at it and go, if they do that, that will achieve my objective so I'm happy to endorse that." – Public Health Consultant*
Find common goals such as equity to bring sectors together	*"And after a few years, my colleagues from the welfare department said there's also a*

	very social aspect in this strategy as well, and we missed that. And we want to be more involved in that part as well. And at that time, we had all these discussions about equity in cities, and so, we said yeah, actually, it's not healthy urban living, but it's healthy urban living for everyone." - International affairs strategist<br><br>*"I find sometimes that the people who choose to work on physical activity or on walking or cycling are people who are personally passionate about cycling or fitness and it's – can be quite unhelpful, because what you want are people who are genuinely passionate about making streets more equitable for people rather than people who just love cycling." – Public Health Consultant*
Develop personal relationships	*"And a lot of it is about not making reasoned evidence based arguments, which is the fallback for the public health community. But really what means people do their jobs differently is politics, personal relationships, a sense of agency over what they're doing, a sense of pride in what they're doing, having the right tools and training available…but I definitely learnt on day one that talking about evidence base gets you pretty much nowhere." - Public Health Consultant*
Develop a governance system	*"So, rather than, you know, people having to keep going to different meetings, there was just one meeting…So, straight away you had a ready-made, sort of, governance system, and I think that's very important, to make sure you've got that governance system. If there isn't one in existence, you have to set it up…And because there isn't a vast army of all these specialists, you know it's going to be the same people over and over again." - Traffic planner*
All stakeholders involved from the beginning	*"And the architects and urban designers were producing the scheme, which will then come to a meeting, which we organised, which had in it local councillors, it had the owners of the site, it had the developers, the potential developers of the site, it had the designers, it had the local planning authority, it had local people, as it were, delegated from a big meeting we held first of all, delegated down through a focus group, so that everyone was there*

	together, and then a particular process of engagement and mutual discussion, to ensure that people started to understand their different points of view." - Emeritus Professor of Planning, Health and Sustainability
**Resource limitations**	
Ask the market for solutions	*"…don't do everything on your own, but try to use each other's strengths, as well. And don't be afraid to ask the market for solutions as well. That was something, one of the best, those bus stops, we were all so excited about it, and it, but particularly because it wasn't our own idea." - International affairs strategist*
When faced with multiple tasks, pick the most impactful ones	*"…people come to you with this long shopping list of things that they want you to do, and you have to – I mean, this is true, I guess, in any job, but you have to stick quite doggedly with doing the few things that you think are going to make a really significant difference, and not get distracted by the things that other people ask you to do. And sometimes when people don't want you to rock the boat too much they will go and ask you to do something that they know will keep you out of the way for a while…So it's picking …asking themselves critical questions about, is this really the best use of my time and am I really being impactful here?" – Public Health Consultant*
Use each other's strengths	*"…don't do everything on your own, but try to use each other's strengths, as well. And don't be afraid to ask the market for solutions as well. That was something, one of the best, those bus stops, we were all so excited about it, and it, but particularly because it wasn't our own idea." - International affairs strategist*
Focus on broader visions to sell to politicians	*"…most often in public administration you look for the money you have and then you say, well we cannot afford a bridge, or we can only afford a very poor one or unpleasant, and so on, and then you use the money you got. But you can turn it the other way round and have it as part of an urban development, and then it's to create visions and ambitions for our politicians, and then they sometimes manage to get the budget for that." – Traffic planner*
Consider starting with a smaller, or easy to sell project.	*"we always think in terms of short, medium, and long term things. And the*

	short ones, which we sometimes call, quick wins, is to have cheap and quick interventions being made that start to show people that things are starting to change, and whether it's some benches somewhere that don't get vandalised, you know"- Architect<br><br>*"And so they inserted the tram system into the heart of the city and they pedestrianized and created cycle routes in the heart of the city. And because pedestrianisation of that was politically easier to do they showed, as it were, showed what was possible. And then basically built out from that with progressive investments in low energy transport, which means walking, cycling, and public transport"- Architect*
**Evaluations**	
Use existing tools and data	*"…try to use available data that's being collected, either as – I mean, there's more and more data collected that's available through things such as, GP practice data, so doctors prescriptions and things like that, that can help looking at before and after, so taking advantage of existing data sources. Or developing approaches so that we can obtain data from individuals on their behaviours through smartphone apps." - Academic*
Trust existing evaluation tools	*"Yeah, the Health and Economic Assessment Tool for walking and cycling, the WHO Tool, so that's all very evidence based…That's great because that's – you've got all the evidence in there - local authority's can use it, and I think they can feed back in the political problems or other problems they have in using it in their locality, and the tool can improve. But it's almost, like, sometimes people are then saying, right now prove that the outcome of that, when you did the lighting, prove that you had the outcome. Well it's all built – the WHO put so much evidence base into the tool you don't need to every time prove the outcome as well, or we're just going round in circles. I mean, the tool needs to be validated and proved and then get on and use it." – Healthy Cities Expert Advisor*
Do evaluations and impact assessments at the beginning or throughout	*"To be very succinct about it, end point evaluations have limited value. They – you might mitigate all the problems but you*

	won't tackle the essence of it. While if you can integrate the process of evaluation right the way through then you've got a real chance of making a good plan, or a good project." - Emeritus Professor of planning, health and sustainability
